# *In vitro* metabolism of synthetic Elabela/Toddler (ELA-32) peptide in human plasma and kidney homogenates analyzed with mass spectrometry and validation of endogenous peptide quantification in tissues by ELISA

**DOI:** 10.1016/j.peptides.2021.170642

**Published:** 2021-11

**Authors:** Duuamene Nyimanu, Richard G. Kay, Rhoda E. Kuc, Alastair J.H. Brown, Fiona M. Gribble, Janet J. Maguire, Anthony P. Davenport

**Affiliations:** a Experimental Medicine and Immunotherapeutics, University of Cambridge, Level 6, Centre for Clinical Investigation, Addenbrooke’s Hospital, Cambridge, UK; b Sosei Heptares, Granta Park, Cambridge, UK; c Metabolic Research Laboratories, Institute of Metabolic Sciences, University of Cambridge, Addenbrooke’s Hospital, Cambridge, CB2 0QQ, UK

**Keywords:** Elabela/Toddler, Apelin receptor, ELA isoforms, ELA-16, Mass spectrometry, Cardiovascular disease

## Abstract

**Background:**

Elabela/Toddler (ELA) is a novel endogenous ligand of the apelin receptor, whose signalling has emerged as a therapeutic target, for example, in cardiovascular disease and cancer. Shorter forms of ELA-32 have been predicted, including ELA-21 and ELA-11, but metabolism and stability of ELA-32 in humans is poorly understood. We, therefore, developed an LC–MS/MS assay to identify ELA-32 metabolites in human plasma and tissues.

**Method:**

Human kidney homogenates or plasma were incubated at 37 °C with ELA-32 and aliquots withdrawn over 2−4 h into guanidine hydrochloride. Proteins were precipitated and supernatant solid-phase extracted. Peptides were extracted from coronary artery, brain and kidney by immunoprecipitation or solid-phase extraction following acidification. All samples were reduced and alkylated before analysis on an Orbitrap mass spectrometer in high and nano flow mode.

**Results:**

The half-life of ELA-32 in plasma and kidney were 47.2 ± 5.7 min and 44.2 ± 3 s, respectively. Using PEAKS Studio and manual data analysis, the most important fragments of ELA-32 with potential biological activity identified were ELA-11, ELA-16, ELA-19 and ELA-20. The corresponding fragments resulting from the loss of C-terminal amino acids were also identified. Endogenous levels of these peptides could not be measured, as ELA peptides are prone to oxidation and poor chromatographic peaks.

**Conclusions:**

The relatively long ELA plasma half-life observed and identification of a potentially more stable fragment, ELA-16, may suggest that ELA could be a better tool compound and novel template for the development of new drugs acting at the apelin receptor.

## Highlights

• Elabela/Toddler (ELA) is the second endogenous ligand of the apelin receptor identified in zebrafish.
• Mature ELA was predicted to be cleaved into [Pyr^1^]ELA-32, ELA-21 and ELA-11, based on the presence of dibasic amino acid residues.
• We have shown that the half-life of [Pyr^1^]ELA-32 was ∼50 min in human plasma and <1 min when incubated with human kidney.
• After incubation of [Pyr^1^]ELA-32 in human plasma, ELA-16 and ELA-11, but not ELA-21, were identified as major metabolites.
• Our study provides information that may lead to the design of biologically stable ELA peptide analogues.

## 1 Introduction

Elabela/Toddler (ELA), encoded by the gene *Apela*, for apelin receptor early endogenous ligand, was discovered independently by two groups in 2013 [1] and 2014 [2], respectively, as the second endogenous ligand of the apelin receptor. ELA was identified in a previously non-coding region of the genome and shown to be essential for proper heart development in zebrafish [1,2]. In zebrafish embryos, ELA deletion resulted in severe cardiac morphogenesis defects and lack of rudimentary heart or no heart at all [1,2]. In adult animals, especially mammals, ELA has been shown to have a role in fluid homeostasis [[3], [4], [5], [6]]. Importantly, administration of ELA is beneficial in several animal models of disease, including pulmonary arterial hypertension [7], heart failure [8] and acute kidney injury [9,10] and therefore ELA signalling may be an important therapeutic target for the treatment of these diseases.

**Fig. 1:**
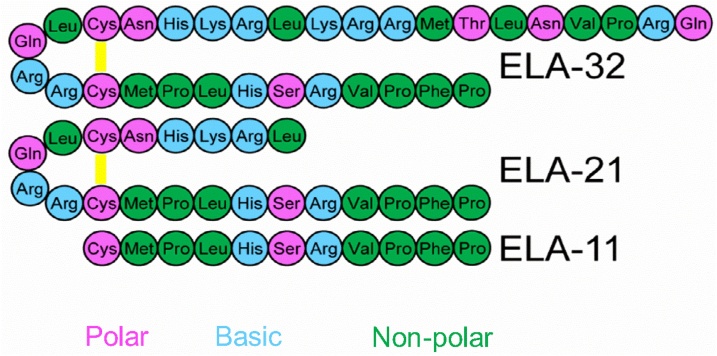
Amino acid sequence of the three predicted ELA isoforms.

ELA is translated as a 54 amino acid preproprotein, which undergoes proteolytic processing to form mature ELA comprising 32 amino acids [1]. Based on the presence of dibasic cleavage sites in the ELA-32 peptide sequence, earlier studies predicted the existence of two other isoforms, ELA-21 and ELA-11 (Fig. 1) [1,2]. We and others have demonstrated that these isoforms bind with high affinity to the apelin receptor and have biological activity [2,7]. The formation of other ELA isoforms, including ELA-22, and ELA-32_(1-9)_, have been reported following incubation of ELA-32 in rodent plasma *in vitro* [4]. To our knowledge, data for similar studies in human plasma and tissues have not been published.

Currently, most efforts to detect and quantify ELA peptides have made use of immunological methods based on the recognition of the shared C-terminal fragment [[11], [12], [13], [14], [15]]. Whilst these methods are very sensitive, they cannot discriminate between ELA isoforms without prior chromatographic separation, as previously reported for apelin isoforms by Maguire et al., [16]. Alternatively, mass spectrometry, also a very sensitive technique, when combined with chromatographic separation, allows for more rapid identification and quantification of individual peptide isoforms. We have, therefore, developed a tandem mass spectrometry-based assay to address the following aims: firstly, to characterise the degradation products generated following incubation of human plasma and tissue with exogenous ELA-32 and secondly, to identify those ELA isoforms that are endogenously produced in human tissues. This information would ascertain the most relevant ELA isoform to more accurately determine the pharmacological and physiological consequences of apelin receptor activation by its second endogenous peptide, ELA.

## 2 Material and method

### 2.1 Materials

[Pyr^1^]ELA-32 (referred to as ELA-32 throughout this paper), ELA-21 and ELA-11 were purchased from Phoenix Pharmaceuticals (Burlingame, USA; Cat No.: 007-19, 007-20 and 007-22, respectively). Human [Pyr^1^]-ELA-32 enzyme immunoassay (EIA) kit (Cat No.: EK-007-19) was purchased from Phoenix Pharmaceuticals (Burlingame, USA). Dynabeads M-280 tosylactivated magnetic beads (Cat. No.: 14203) were from Invitrogen (California, USA), and Protein A/G magnetic beads (Cat No.: 17152104011150) were sourced from GE Healthcare (Chicago, USA). Protein LoBind Eppendorf tubes (Cat No.: 0030108094) and Protein LoBind 96-well Deepwell plates (Cat No.: 0030504216) were from Eppendorf (Stevenage, UK), and Oasis Prime μ-Elution 96-well plates (Cat No.: 186008052) from Waters (Wilmslow, UK). Iodoacetamide (Cat. No.: I1149-25 G), 1,4-Dithiothreitol (DTT) (Cat No.: 43819-5 G), ammonium bicarbonate (Cat. No.: A-6141), acetonitrile (ACN) (Cat. No.: 270717) and glacial acetic acid (Cat. No.: 33209-1 L) were purchased from Sigma-Aldrich (Gillingham, UK), now Merck; methanol (Cat. No.: 10675112) and 0.1 % formic acid in water (Cat. No.: LS118-212) from Fisher Scientific (New Hampshire, USA) and guanidine hydrochloride (6 M) solution (Cat. No.: B1013-1 L) was from BioVision (Milpitas, USA).

Surgical samples of human tissue were obtained with informed consent from Royal Papworth Hospital Research Tissue Bank and ethical approval (05/Q104/142) and Cambridge Brain Bank for tissue supported by the NIHR Cambridge Biomedical Research Centre.

### 2.2 ELA metabolism in human plasma and human kidney homogenate

Blood from four healthy human donors (3 males, 1 female) was centrifuged (2,000 x*g*, 5 min, 4 ⁰C), and the resulting plasma pooled. To assess ELA metabolism in human kidney homogenate, 2 g of human kidney from three individuals were pooled and homogenised in 50 mM HEPES solution, pH 7.4, using a polytron probe with 3 x 20 s bursts. Both human plasma and homogenate samples were stored at −70 °C until needed. For the assay, human plasma (1.2 mL) and kidney homogenate (1.8 mL) samples were maintained at 37 °C throughout. After 25 min, plasma (50 μL) and kidney (100 μL) samples were withdrawn as blank controls. ELA-32 (final concentration 5 μg/mL) was then added (time 0) and further samples were withdrawn at different time-points (plasma: 0, 2, 5, 10, 15, 30, 60, 120, 240; homogenate: 0, 2, 5, 10, 15, 30, 60, 120) and mixed with half volume ice-cold guanidine hydrochloride (6 M) to terminate enzyme reactions. Proteins were precipitated with 75 % ACN in water, centrifuged (12,000 xg, 5 min, 4 ⁰C) and supernatants were extracted using the solid-phase extraction (SPE) method described below. Following reduction and alkylation (see Section 2.3), the resulting samples were analysed by LC–MS/MS in both high flow and nano flow configuration.

### 2.3 Reduction and alkylation

The peptide extracts were reconstituted in ammonium bicarbonate (75 μL, 50 mM) containing 10 mM 1,4-dithiothreitol (DTT) and, in initial experiments, heated to 60 ⁰C for 60 min. This was subsequently reduced to 45 min to minimise side reactions. A 15 μL aliquot of iodoacetamide (100 mM) prepared in 50 mM ammonium bicarbonate was added and samples incubated in the dark for 30 min at room temperature. The reaction was stopped by addition of DTT (20 μL, 10 mM) in the light, followed by 20 μL of 1% formic acid in water (v/v).

### 2.4 High flow rate LC–MS/MS analysis

The initial metabolite identification in the extracted plasma and kidney samples was performed using an Ultimate 3000 LC system coupled to a Q-Exactive Plus Orbitrap (both Thermo Scientific, San Jose, USA) using the HESI-plus source interface. An Acquity HSS T3 C18 2.1 × 50 mm column (Waters, Wilmslow, UK) was used for separation of the peptides and was held at a temperature of 60 °C. Mobile phases used for separation were A: 0.1 % formic acid, 0.01 % trifluoroacetic acid (TFA) in water, and B: 0.1 % formic acid, 0.01 % TFA in ACN, where initial conditions were 2 % B at a flow rate of 300 μL per minute. Peptides were eluted over a 16-minute gradient where the %B was raised to 45 %, followed by a 2-minute column clean at 90 % B before returning to initial conditions with a total of 20-minute run. Full scan data was collected from 400 to 1600 *m/z* with a resolution of 70,000. A total of 10 μL of the extract was injected for each analysis.

### 2.5 Nano flow LC–MS/MS analysis

Peptide extracts were all analysed on the same LC–MS system in nano flow mode, with the same mobile phases containing 0.01 % TFA. Extracts (30 μL) were loaded onto a 0.3 × 5 mm peptide trap column (Thermo Scientific, Waltham, USA) at a flow rate of 30 μL/min using 2% B, and washed for 15 min before switching in line with a 0.075 × 250 mm nano easy column (Thermo Scientific, Waltham, USA) running at 300 nL/min. Both nano and trap column temperatures were set at 45 °C. Initial conditions were 2% B for 15 min. A ramp to 50 % B was performed over 90 min and the column was then washed with 90 % B for 20 min before returning to starting conditions for a further 20 min, totalling an entire run time of 130 min. Positive nanoelectrospray analysis was performed using a spray voltage of 1.8 kV; the tune settings for the mass spectrometer used an S‐lens setting of 70 V to target peptides of higher *m/z* values. A full scan range of 400–1600 *m/z* was performed at a resolution of 75,000 before the top 10 ions of each spectrum were selected for MS/MS analysis. Exiting ions selected for fragmentation were added to an exclusion list for 30 s.

A targeted parallel reaction monitoring method was built to specifically target ELA peptides for quantitative analysis and to facilitate detection of endogenous peptides. This was performed in a single method in combination with an information-dependent acquisition (IDA) analysis, where the instrument targeted the highest abundant charge state for ELA-32 (*m/z*: 586.16, 7+ and 581.88, 7+), ELA-21 (*m/z*: 451.39, 6+) and ELA-11 (*m/z*: 447.55, 3+).

### 2.6 Method development for analysis of endogenous ELA in human tissues

Various extraction methods and LC–MS/MS approaches were employed to enhance the detection of endogenous ELA peptides in human tissues. Firstly, initial experiments were performed using kidney, and coronary artery as these tissues were reported to express the highest mRNA levels of ELA [1,3,7]. Secondly, following a lack of detection using the guanidine hydrochloride method, bead-based immunoprecipitation of ELA after extraction was performed with the aim of increasing sensitivity. For this study, we included human normal brain (control), where low levels of ELA peptide was expected and glioblastoma (GBM) samples in which ELA levels were previously shown to be significantly higher [17].

LC–MS analysis of endogenous ELA peptides proved extremely challenging. The physicochemical properties of these peptides (highly charged due to multiple arginine and lysine residues) resulted in high interaction with the solid phase material, creating extremely broad peaks using the usual peptide LC–MS reagents and consumables. ELA-32 and ELA-21 showed particularly poor chromatographic separation characteristics using formic acid as a mobile phase modifier and required the addition of TFA to obtain acceptable peaks.

#### 2.6.1 Peptide using guanidine hydrochloride

Peptides were extracted as previously described [18]. In brief, approximately 25 mg human kidney (n = 5) and 50 mg human coronary artery (n = 7) were transferred into Lysing MatrixD ceramic beads tubes. A mixture of ELA-32, ELA-21 and ELA-11 (each at 100 ng/mL) with kidney (˜10 mg) as a matrix was used as a positive control. Guanidine hydrochloride (500 μL, 6 M) was added to each tube and tissues homogenised using the FastPrep-24TM 5 G system (MP Biomedicals, Santa Ana, USA) for 4 runs (6.0 m/s, 40 s) and centrifuged (1000 xg, 5 min, 4 ⁰C). A 200 μL aliquot of homogenate was transferred to protein LoBind tubes containing 800 μL aliquot of 80 % (v/v) ACN in deionised water and centrifuged (12,000 xg, 5 min, 4 ⁰C). The organic phase was discarded, and the aqueous (second phase) containing the peptides transferred to a 96-well protein LoBind plate and evaporated under oxygen-free nitrogen at 40 ⁰C on a Biotage SPE dry (Upsala, Sweden). Samples were reconstituted in 500 μL 0.1 % formic acid in water, vortex-mixed and transferred to 96-well Oasis Primed HLB μ-Elution plate and extracted in a positive pressure SPE manifold (Waters, Wilmslow, UK). After washing with 200 μL 5% methanol:1% acetic acid in water, peptides were eluted with 2 × 30 μL 60 % methanol:10 % acetic acid (v/v) in water, centrifuged (1200 rpm, 2 min, RT) and dried under oxygen-free nitrogen. Peptides were subsequently reduced and alkylated and stored at −20 ⁰C until analysis on LC–MS/MS in the nano flow mode (see Section 2.5 above).

#### 2.6.2 Peptide extraction and immunoprecipitation

Human kidney (˜250 mg), coronary artery (˜150 mg), normal brain (˜300 mg) and GBM (˜300 mg) samples were transferred into Lysing MatrixD ceramic bead tubes and stored at −70 °C until needed. Boiling water (1 mL, 100 °C) was added to each tube as previously described [2]. Samples were boiled for 20 min, cooled to room temperature, homogenised using the FastPrep-24TM 5 G system (MP Biomedicals, USA) for three runs (6.0 m/s, 40 s) and centrifuged (1000 xg, 5 min, 4 °C) to obtain extracted peptides in the supernatant. The resulting samples were stored at −70 °C until required.

For immunoprecipitation of ELA, samples containing extracted peptides were transferred to a protein Lobind tube containing antibody-coated beads. A mixture of ELA-32, ELA-21 and ELA-11 (each at 100 ng/mL) was added to a tube containing 0.1 % bovine serum albumin (BSA) used as surrogate matrix and 10 μL ELA-32 antibody (1 mg/mL) as a positive control. All samples were then incubated at 4 °C overnight with end-to-end rotation. Following a 2 × 500 μL wash in deionised water, peptides were eluted by incubating beads with 100 μL of 60 % methanol:10 % acetic acid in water (v/v) for 20 min with end-to-end rotation. An 8-point calibration curve (10 pg/mL-50,000 pg/mL) was generated in 0.1 % BSA used as surrogate matrix from a stock solution of ELA-32, ELA-21 and ELA-11 (each at 10 μg/mL). The eluted peptides and 150 μL of each standard were evaporated under oxygen-free nitrogen at 40 °C.

#### 2.6.3 Peptide extraction using guanidine hydrochloride and TFA

Peptide samples (500 μL), prepared as described for immunoprecipitation (Section 2.6.2 above), were further extracted with guanidine hydrochloride (250 μL, 6 M), precipitated with 75 % ACN in water (v/v) and centrifuged (12000 xg; 5 min, 4°C) before the supernatant was transferred to a fresh tube. The remaining pellet was reconstituted in 0.5 % TFA in water (v/v) to ion-pair with any highly positive ELA peptide that may have remained in the sample, centrifuged (12000 xg; 5 min, 4°C), and the supernatant transferred to a fresh tube. All samples were then dried down and solid-phase extracted as previously described (see Section 2.6.1 above). Samples were pooled, evaporated to dryness, reduced and alkylated (see Section 2.3 above) before analysis (20 μL) by LC–MS/MS in nano flow mode.

### 2.7 Peptide identification using PEAKS software

LC–MS/MS data were interrogated using the PEAKS X software (BSI, Waterloo, Canada) against the human Uniprot database (downloaded 18-11-2018) as well as a custom database containing only the ELA peptide using a no-enzyme setting. PEAKS software *de novo* sequences mass spectra before running the resulting peptide sequences against protein sequences in the UniProt protein database [19]. Where extracts had been reduced and alkylated, a fixed carbamidomethylation modification was applied to cysteine residues. Variable changes included N-terminal acetylation, N-terminal pyroglutamination, C-terminal amidation and methionine oxidation. After initial analyses, it was discovered that in some cases, the ELA peptide became over alkylated, where lysine residues acquired additional alkylations (+57.02 Da), and therefore this was added as another variable modification. An FDR setting of 1% was used against a decoy database, and the precursor and product ion tolerances were set as 10 ppm and 0.05 *m/z*, respectively.

### 2.8 Enzyme immunoassay

Peptides extracted from kidney and coronary artery, analysed by LC–MS/MS to detect and quantify ELA isoforms, were subsequently evaporated to remove formic acid and reconstituted in assay buffer for re-analysis using an ELISA. This assay cross-reacts with known isoforms of ELA including [pGlu^1^]ELA-32, ELA-21 and ELA-11 [7] and has an inter- and intra-assay variability of <15 % and <10 %, respectively, with a minimum detectable concentration of 0.19 ng/mL. The ELISA assay was performed following the manufacturer’s instructions. Briefly, duplicate samples were added to wells previously coated with a secondary antibody. The primary antibody and biotinylated peptide were subsequently added and incubated with rotation (300−400 rpm) for 2 h. After washing and blot drying (four times), streptavidin-conjugated horseradish peroxidase was added and incubated for 1 h with rotation. The substrate solution tetramethylbenzidine was added after another round of washes and blot drying (four times) and incubated for a further 1 h. The reaction was quenched by the addition of 2 N HCl and absorbance measured at 450 nm using a Synergy HT microplate reader (Biotek, Vermont, USA). The absorbance was proportional to the amount of biotinylated peptide–peroxidase complex and, therefore, inversely proportional to the concentration of peptides in the samples. Unknown concentrations were determined by interpolation to a standards curve fitted to a 4-parameter logistic concentration-response curve in GraphPad Prism 6 (La Jolla, USA).

### 2.9 Data analysis

Peak areas were quantified using the Quanbrowser software (Thermo Scientific, Waltham, USA) and expressed as mean ± SD. ELA levels determined by ELISA were defined as mean ± SEM.

## 3 Results

### 3.1 ELA-32 is more stable in human plasma than kidney

**Fig. 2:**
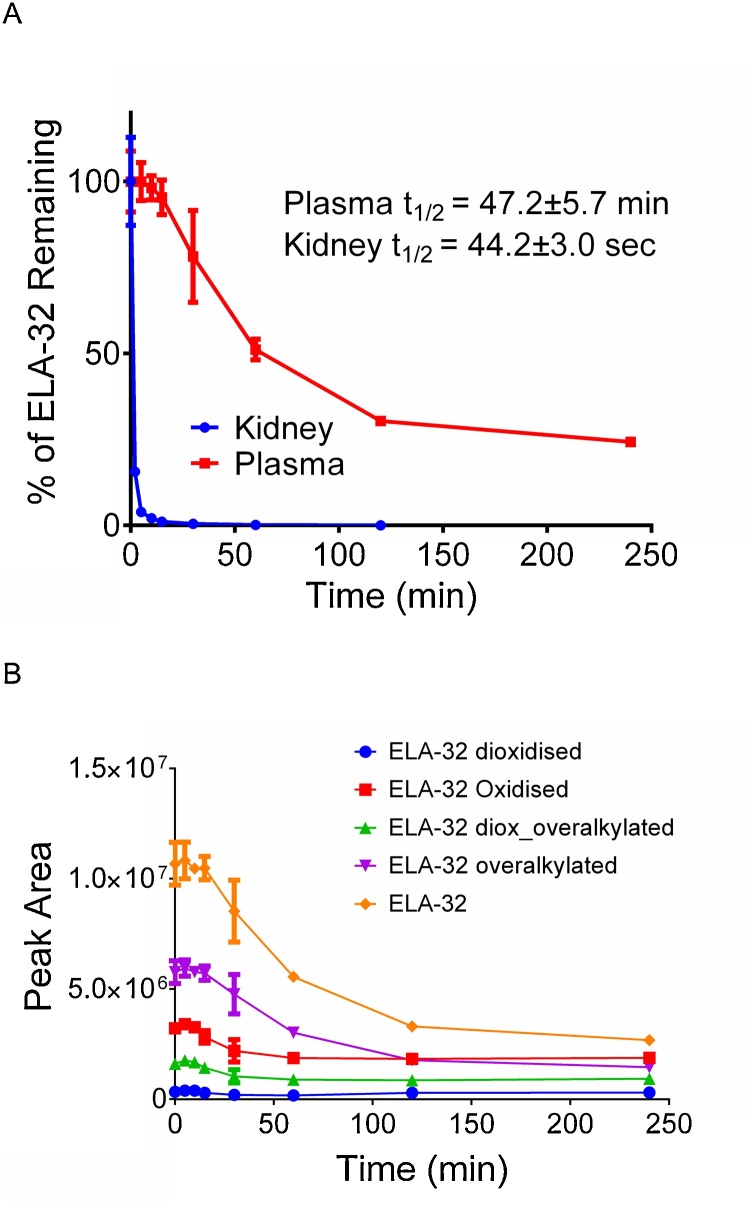
Stability of ELA-32 in human plasma and kidney homogenates. A, Half-life of ELA-32 in human plasma and kidney homogenates, B, ELA-32 showing some of the side reactions observed in human plasma. Data represent mean ± SD.

ELA-32 peptide degraded relatively slowly in human plasma such that over 20 % of the parent peptide was still detectable after 240 min (Fig. 2A). However, degradation in the kidney was very rapid, with the parent peptide undetectable after 20 min (Fig. 2A). The half-life of ELA-32 in plasma and kidney was 47.2 ± 5.7 min and 44.2 ± 3 s, respectively. In addition to the expected cysteine alkylation, several other modifications of the parent ELA-32 peptide were apparent, including mono-oxidation, di-oxidation and alkylation of lysine residues (referred to as over-alkylation throughout this article) (Fig. 2B), likely to represent extraction artefacts.

### 3.2 Identification of *in vitro* metabolites of synthetic ELA-32 peptide

In order to identify metabolites from ELA-32 in human plasma and tissue homogenates, a full scan analysis was first performed to understand the degradation profile of intact ELA-32 peptide in the high flow LC–MS/MS. However, subsequent database searching failed to identify any metabolites, as no suitable product ion spectra were generated using the rapid analysis. Therefore, selected samples were re-analysed using the more sensitive nano LC–MS/MS and a data-dependent acquisition (DDA) analysis. The LC–MS/MS data were analysed on PEAKS X software and compared against the UniProt protein database. The amino acid sequences, observed mass and mass accuracy (ppm) for the C-terminal and N-terminal metabolites of ELA-32 that were identified are shown in Table 1, Table 2.

**Table 1:** ELA-32 fragments derived from loss of C-terminal amino acid residues.

	C-terminal metabolites	Length	Mass	ppm	*m/z*	Charge
ELA-32	QRPVNLTMRRKLRKHNCLQRRCMPLHSRVPFP	1-32	4096.16	0.8	513.03	8
	**C-terminal metabolites**					
ELA-32_(1-31)_	QRPVNLTMRRKLRKHNCLQRRCMPLHSRVPF	1-31	3967.12	9.2	441.80	9
ELA-32_(-21)_	QRPVNLTMRRKLRKHNCLQRR	1-21	2758.53	0.4	460.76	6
ELA-32_(1-20)_	QRPVNLTMRRKLRKHNCLQR	1-20	2602.43	1.2	434.74	6
ELA-32_(1-19)_	QRPVNLTMRRKLRKHNCLQ	1-19	2487.35	−0.3	415.57	6
ELA-32_(1-16)_	QRPVNLTMRRKLRKHN	1-16	2045.15	0.9	410.03	5
ELA-32_(1-14)_	QRPVNLTMRRKLRK	1-14	1835.08	0.9	459.78	4
ELA-32_(1-13)_	QRPVNLTMRRKLR	1-13	1665.96	−0.8	417.49	4
ELA-32_(1-12)_	QRPVNLTMRRKL	1-12	1509.85	−0.1	504.29	3

	**Other Metabolites generated**					
ELA-19_(1-17)_	KHNCLQRRCMPLHSRVP	13-30	2204.10	3.1	1103.06	2
ELA-16_(1-14)_	CLQRRCMPLHSRVP	16-30	1824.90	0.3	457.23	4

ELA-19_(1-17)_ and ELA-16_(1-14)_ were derived from des-Pro^17^-ELA-19 and des-Pro^16^-ELA-16, respectively and maybe a substrate of ACE2.

**Table 2:** ELA-32 fragments produced from loss of N-terminal amino acid residues.

	N-terminal metabolites	Length	Mass	ppm	*m/z*	charge
ELA-32	QRPVNLTMRRKLRKHNCLQRRCMPLHSRVPFP	32	4096.2	0.8	513.03	8
ELA-28_(5-32)_	NLTMRRKLRKHNCLQRRCMPLHSRVPFP	5-32	3673.9	−1.1	525.86	7
ELA-23^ͳ^_(9-32)_	RKLRKHNCLQRRCMPLHSRVPFP	9-32	3042.6	0.5	508.11	6
ELA-22_(10-32)_	KLRKHNCLQRRCMPLHSRVPFP	10-32	2845.5	2	475.26	6
**ELA-20_(12-32)_**	**RKHNCLQRRCMPLHSRVPFP**	**12-32**	**2604.3**	**0**	**435.06**	**6**
**ELA-19_(13-32)_**	**KHNCLQRRCMPLHSRVPFP**	**13-32**	**2448.2**	**0.2**	**490.65**	**5**
**ELA-18_(14-32)_**	**HNCLQRRCMPLHSRVPFP**	**14-32**	**2320.1**	**0.8**	**465.03**	**5**
ELA-17_(15-32)_	NCLQRRCMPLHSRVPFP	15-32	2183.1	−0.2	437.62	5
**ELA-16_(16-32)_**	**CLQRRCMPLHSRVPFP**	**16-32**	**2069.0**	**0.6**	**414.81**	**5**
**ELA-11_(21-32)_**	**CMPLHSRVPFP**	**21-32**	**1355.6**	**0.3**	**452.89**	**3**
ELA-9_(23-32)_	PLHSRVPFP	23-32	1048.6	2.2	525.30	2
ELA-8_(24-32)_	LHSRVPFP	24-32	951.53	1.3	476.77	2

Mass, observed mass; ppm, parts per million (is a measure of mass accuracy); *m/z*, mass to charge ratio. The most important isoforms are shown in bold. **^ͳ^**this metabolite was low plasma but very high in tissue homogenate.

#### 3.2.1 *In vitro* metabolism of synthetic ELA-32 peptide in human plasma and tissue

**Fig. 3:**
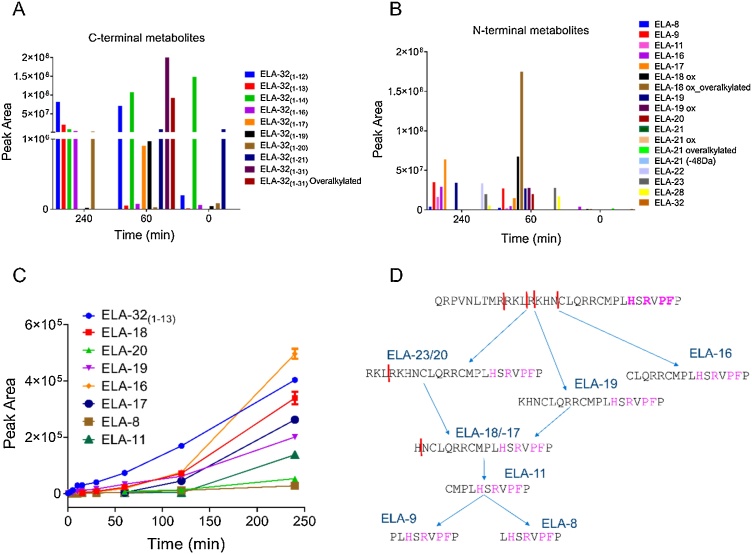
ELA-32 metabolites identified from human plasma following nano and high flow LC–MS/MS analysis. A, C-terminal metabolites generated as a product of cleavages at the C-terminus of ELA-32; B, N-terminal metabolites generated as a product of cleavages at the N-terminus of ELA-32. C, Metabolites identified from manual searching of high flow data. D., schematic representation of ELA-32 degradation profile. Coloured (magenta) amino acid residues were previously shown to be critical for receptor binding [4].

**Fig. 4:**
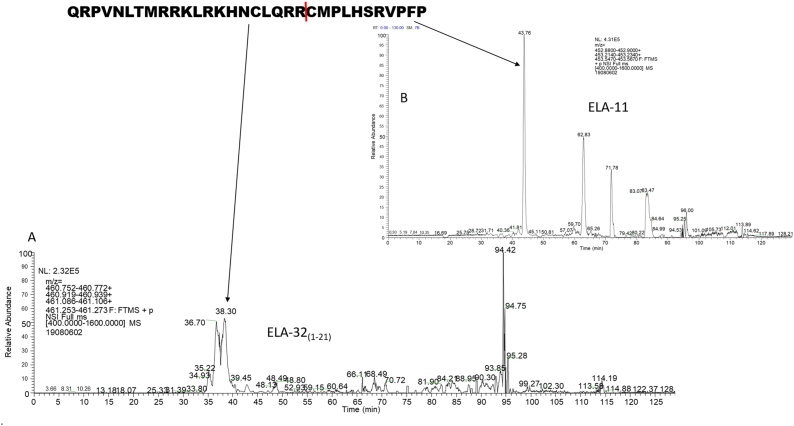
Representative Chromatogram of ELA-32_(1-21)_ (A) and ELA-11 (B) generated from ELA-32. The metabolites were produced from the hydrolysis of mature ELA-32 between Arg^21^ and Cys^22^, but the hydrolytic enzyme is unknown. ELA-32_(1-21_*_)_*, retention time 38.30 min; ELA-11, retention time 43.76 min, was only present in the oxidised form (methionine oxidation). The red line indicates the cleavage point.

**Fig. 5:**
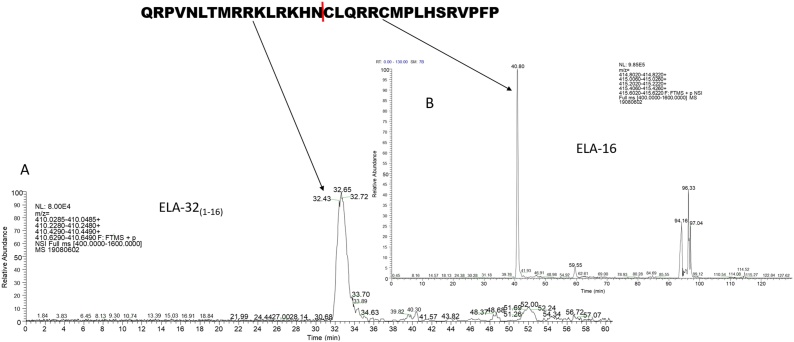
Representative chromatogram of ELA-32_(1-16)_ (A) and ELA-16 (B) produced from the hydrolysis of ELA-32. ELA-32_(1-16)_ and ELA-16 were generated from the cleavage between Asn^16^ and Cys^16^. ELA-32_(1-16)_, retention time 32.65 min; ELA-16 was only present in the oxidised form (methionine oxidation); retention time 40.80 min. The red line indicates the cleavage point.

In plasma, ELA-32 was cleaved to produce both N- and C-terminal metabolites. The most abundant metabolites produced from the loss of C-terminal amino acids (C-terminal metabolites) were ELA-32_(1-31)_, ELA-32_(1-14)_ and ELA-32_(1-12)_ (Table 1; Fig. 3A). The corresponding C-terminal fragments of some of these metabolites (N-terminal metabolites) were also identified, including ELA-18, ELA-20 and ELA-11 (Table 2; Fig. 3B). ELA-16, ELA-19 and ELA-22 were also present, but there was no evidence of ELA-21, previously thought to be produced from ELA-32 [1]. Interestingly, ELA-16_(1-14)_ and ELA-19_(1-17)_ generated from cleavage between the C-terminal proline and phenylalanine residue of the corresponding des-Pro^16^-ELA-16 and des-Pro^19^-ELA-19 peptides, respectively, were identified, which is suggestive of ACE2 activity (Table 2). Chromatograms of the most relevant metabolites, ELA-11 (previously proposed as a metabolite of ELA-32), and ELA-16 (because of the disulphide bridge which will stabilise it against proteolysis), are shown in Fig. 4, Fig. 5, respectively. The chromatogram of all other metabolites is shown in supplementary Figs. 1A-G.

##### 3.2.1.1 Metabolism profile of ELA-32 in human plasma

In order to elucidate the sequential metabolite generation profile, the original full-time course dataset from the high flow full scan LC–MS/MS was re-analysed and interrogated for metabolites by manual searching for the theoretical mass of the peptides identified in the nano LC–MS/MS analysis. A complication was that the nano flow analysis generated higher charge states for most of the identified peptides as it is more efficient than standard flow, and the TFA reduced the efficiency further. The data showed that within 2 min, ELA-16 and ELA-19 could be detected, with ELA-8 and ELA-18 generated from 5 and 15 min, respectively. All other fragments resulting from the loss of N-terminal amino acids were detected from 60 min, when the most abundant metabolites were ELA-20, ELA-17, and ELA-11 (Fig. 3C).

By the end of the incubation period (240 min), the most abundant metabolites were ELA-16, ELA-18, ELA-17, ELA-19 and ELA-11 in descending order. The N-terminal fragment, ELA-32_(1-13)_, resulting from the cleavage that produced ELA-19, was also observed at the same time as ELA-19. We were unable to detect ELA-9, ELA-21, ELA-22, ELA-23 and ELA-28 by this method (Fig. 3C). Taken together, these analyses showed that ELA-32 was initially cleaved to ELA-19, ELA-16 and possibly ELA-23 and ELA-20. These fragments were subsequently cleaved into smaller fragments (Fig. 3D). Interestingly, the four C-terminal amino acid residues previously shown to be critical for receptor binding [4] were not affected. Therefore, the most important and biologically relevant isoforms identified in human plasma were ELA-20, ELA-19, ELA-16 and ELA-11.

#### 3.2.2 *In vitro* metabolism of synthetic ELA-32 peptide in kidney lysates

**Fig. 6:**
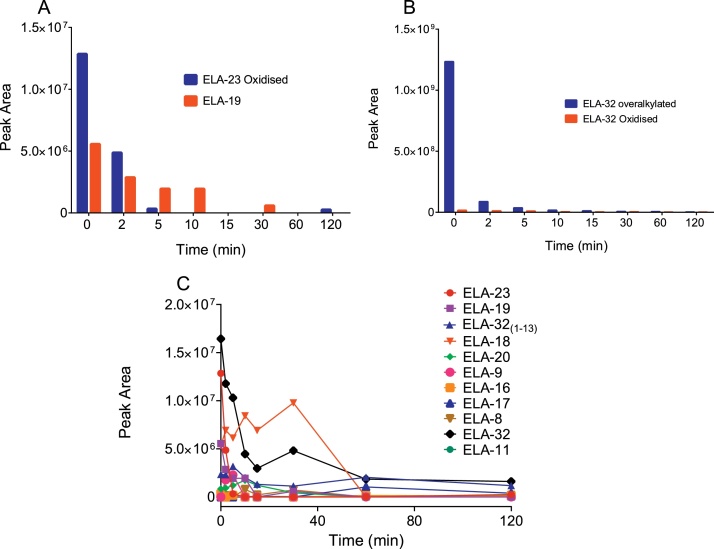
ELA-32 metabolites identified from human kidney homogenates following nano and high flow LC–MS/MS analysis. A, Metabolites generated as a product of cleavages at the N-terminus of ELA-32 identified by nano flow analysis; B, levels of modified ELA-32 peptides; C, Metabolites identified from manual searching of fast-flow data. No C-terminal metabolites were identified in this case.

In the nano flow analysis, only two fragments resulting from the loss of N-terminal amino acids, namely ELA-23 and ELA-19, were identified in the kidney (Fig. 6A). Interestingly, we also identified ELA-32_(1-13)_, the N-terminal sequence removed from ELA-32 to generate ELA-19 through manual searching. The parent peptide levels decreased rapidly and were present in oxidised and overalkylated forms (Fig. 6B).

The high flow LC–MS/MS data were re-analysed and searched for the same metabolites that were identified in plasma. However, unlike plasma, where ELA-16 was most abundant, ELA-18 was highest in the kidney (Fig. 6C). In addition, ELA-23, ELA-19 and ELA-16 were detected from the earliest time points. However, their levels decreased significantly with time, except for ELA-18, whose level was highest 30 min after incubation but was completely undetectable after 60 min. ELA-17 was only detected in the last one hour of the incubation (60 min and 120 min samples), with the lowest level present at the latter time point. However, there was no evidence of ELA-21 in these samples, but ELA-11 was present across all time points. Taken together, the most important isoforms identified in human kidney were ELA-23, ELA-19, ELA-18, ELA-16 and ELA-11.

### 3.3 Development of extraction methods for detection of endogenous ELA isoforms by LC–MS/MS

Having identified potential endogenous cleavage sites of ELA-32 *in vitro*, we next aimed to identify the most abundantly produced endogenous peptide. As it was previously shown that the highest level of ELA mRNA expression was present in adult human coronary artery [7] and kidney [1,3], peptides were therefore extracted from these tissues using the guanidine hydrochloride-based method previously described by Roberts et al. [18]. The resulting nano flow LC–MS/MS analysis followed by database search with PEAKS software was unable to find any evidence for endogenous ELA peptides in these tissues.

**Fig. 7:**
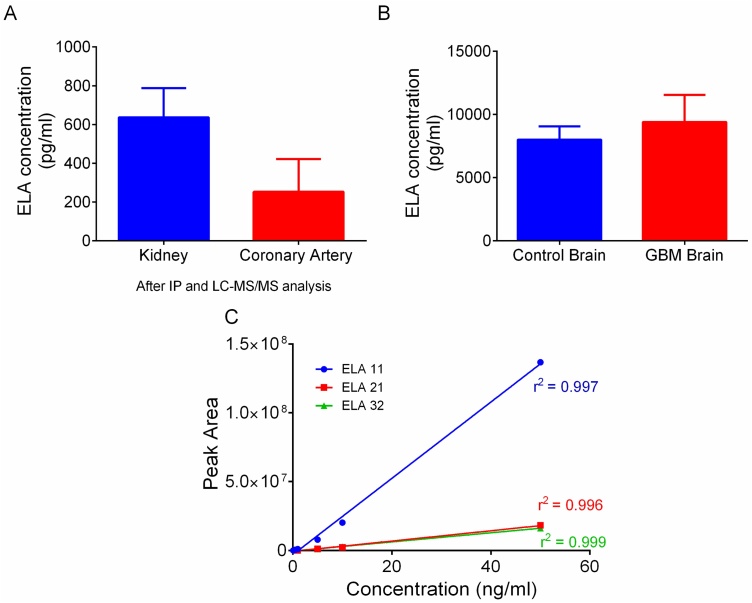
Analysis of endogenous ELA peptides in human tissues. A, ELA levels in the kidney and coronary artery samples measured by ELISA after immunoprecipitation and LC–MS/MS analysis. B, ELA concentration in control (normal) brain compared to GBM samples measured by ELISA. C, ELA standards calibration line used to determine instrument sensitivity for each ELA isoforms on the LC–MS/MS system; ELA-11 r^2^ = 0.997, ELA-21 r^2^ = 0.996, ELA-32 r^2^ = 0.999. Data represent mean ± SEM.

Antibody-based enrichment of peptides is a well-characterised method for concentrating analytes in solution [20]. Therefore, we first showed that ELA-antibody coated magnetic beads were capable of extracting ELA-32, ELA-21 and ELA-11 (Supplementary Fig. 2). Extracted peptides were then immunoprecipitated with ELA antibody-coated magnetic beads and analysed on the nano flow LC–MS/MS, but no ELA peptides were detected in either a human coronary artery or kidney samples (data not shown). To further validate these results, extracts were re-analysed by ELISA after LC–MS/MS. Interestingly, the levels of ELA present in the kidney samples after mass spectrometry were 637.1 ± 151 pg/mL, while that of the coronary artery was 253.2 ± 168 pg/mL (Fig. 7A). Because the levels present in coronary artery were deemed too low, it was removed from subsequent studies while normal brain and samples obtained from GBM patients were added. ELISA showed that ELA levels in both normal and GBM brain were very high, 8013.0 ± 1041 pg/mL *vs* 9410.0 ± 2139 pg/mL, respectively (Fig. 7B).

Pauli et al., 2014 [2] previously extracted ELA peptides by boiling in water, followed by acidification before the resulting samples were concentrated (pooled together). This method allowed detection of ELA-11 endogenously by LC–MS/MS in their over-expression model, but none of the longer isoforms was detected. To increase the chance of detecting endogenous ELA peptides, human kidney, normal brain and GBM samples were extracted using the above method. Samples were then pooled together with the aim of increasing the concentration of ELA present. In parallel, some samples were also immunoprecipitated as described earlier, and a mixture of ELA-11, ELA-21 and ELA-32 in 0.1 % BSA (surrogate matrix) at 100 ng/mL used as a positive control. An 8-point calibration curve (10 pg/mL – 50 ng/mL) was constructed for each ELA isoform to determine the lower limit of detection (LLOD). ELA-11 calibration line (r^2^ = 0.997) showed that the LLOD for this peptide was 100 pg/mL. However, the LLOD for the longer isoforms ELA-21 (calibration line r^2^ = 0.996) and ELA-32 (calibration line r^2^ = 0.999) was five-fold higher at 500 pg/mL (Fig. 7C).

**Fig. 8:**
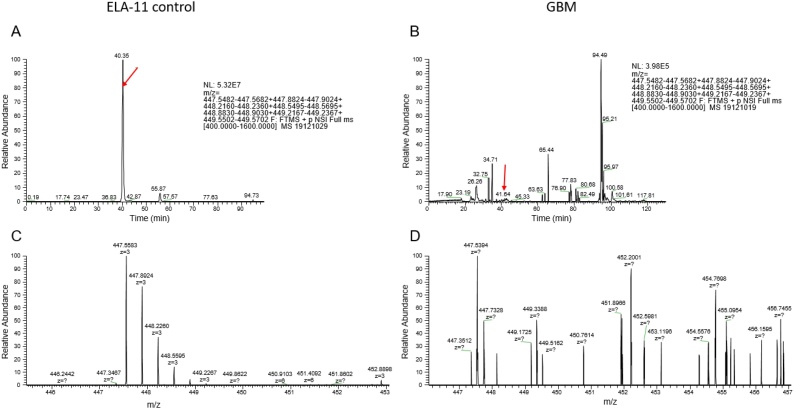
Representative chromatograms of ELA-11 peptides detected endogenously in human tissues. ELA-11 positive control chromatogram (A) and its carbon-13 isotopes (C); B, D; endogenous ELA-11 chromatogram and its carbon-13 isotopes (D).

Full scan analysis was performed in parallel to the targeted analysis. However, none of the ELA peptides appeared to be present (Fig. 8). Furthermore, targeted analysis of the samples for ELA-32 (*m/z*: 586.16, 7+ and 581.88, 7+), ELA-21 (*m/z*: 451.39, 6+) and ELA-11 (*m/z*: 447.55, 3+), was unable to detect any of the peptides in the extracts. This suggests that if the peptides are present in these tissues, then they are below the level of detection of the developed LC–MS method.

A database search of the raw LC–MS/MS data failed to identify any ELA peptides in any of the samples; however, several neuropeptides differentially produced/expressed in the control and GBM brain were detected. This included Cocaine- and amphetamine-regulated peptide, neuropeptide Y, cholecystokinin and Pro-SAAS family of peptides (Table 3).

**Table 3:** Selected key peptides present in control (normal) brain but not GBM.

Protein	Sequence	−10logP	Mass	Length	ppm	m/z	RT
**ProSAAS Peptides**							
GAV	SVPRGEAAGAVQELARALAHLLEAERQE	58.37	2970.564	28	1.2	743.649	72.63
PEN (mouse)/PEG (Human)	AADHDVGSELPPEGVLGALL	50.86	1958.995	20	0.3	980.505	70.65
Little SAAS	GLSAASPPLAETGAPRRF	43.71	1796.953	18	−1.4	599.9908	44.21
SAAS	ARPVKEPRGLSAASPPLAETGAPRRF	39.13	2730.504	26	1.4	547.1089	39.49

**Cholecystokinn Peptides**							
	QPVPPADPAGSGLQRAEEAPRRQL	48.67	2522.299	24	−0.7	841.7729	45.78
	QPVPPADPAGSGLQRAEEAPRRQ	46.89	2409.215	23	−1.6	804.0776	41.2
	ADPAGSGLQRAEEAPRRQL	45.43	2021.04	19	−2	1011.525	35.43
	DPAGSGLQRAEEAPRRQL	32.08	1950.003	18	−2.1	651.007	35.68

**Secretogrannin-1 Peptides**							
	QYDRVAQLDQLLHY	47.53	1743.858	14	1.4	872.9375	65.21
	NLARVPKLDL	41.77	1137.687	10	0.1	569.8508	46.71
	KNFFPEYNY	20.44	1220.55	9	4.8	611.2853	51.56

**Secretogrannin-2 Peptides**							
	TNEIVEEQYTPQSLATLESVFQELGKLTGPNNQ	53.6	3676.811	33	−0.1	1226.611	83.6
	FPVGPPKNDDTPNR	40.36	1552.763	14	0.3	777.3892	34.63
	FPVGPPKNDDTPNR	19.73	1609.785	14	0.5	537.6025	34.56
	GQGSSEDDLQEEEQIEQAIKEHLNQGSSQETDKLAPVS	16.71	4152.92	38	2.2	1385.317	58.72

**Secretogrannin-3 Peptides**							
	ELSAERPLNEQIAEAEED	49.36	2041.944	18	1.3	1021.981	47.64

**Cocaine- and amphetamine-regulated transcript**							
	ALDIYSAVDDASHEKELIEALQEVLKKL	58.27	3139.665	28	1.2	628.941	79.87

**Neurogrannin Peptides**							
	ARKKIKSGERGRKGPGPGGPGGAGVARGGAGGGPSGD	32.75	3312.762	37	0	474.259	26.77
	PGANAAAAKIQASFRGHMARKKIKSGERGRKGPGPGGPGGAGVARGGAGGGPSGD	21.4	5148.684	55	−0.1	736.5334	32.05
	DPGANAAAAKIQASFRGHMARKKIKSGERGRKGPGPGGPGGAGVARGGAGGGPSGD	16.42	5263.71	56	1.3	658.9719	32.91
	PGANAAAAKIQASFRGHMARKKIKSGERGRKGPGPGGPGGAGVARGGAGGGPSGD	14.08	5148.684	55	1.5	644.5937	32.05

**Neuropeptide Y**							
	SPETLISDLLMRESTENVPRTRLEDPAMW	85.85	3472.7	30	1	1158.574	60.45
	YPSKPDNPGEDAPAEDMARYYSALRHYINL	80.68	3452.61	30	0.1	864.16	54.8

-10logP = measure of confidence in sequence matching; ppm = parts per million; *m/z* = mass to charge ratio; RT = retention time; length refers to peptide length detected. The peptides identified were CCK-18, CCK-19, CCK-23 and CCK-24.

## 4 Discussions

In this study, we have developed an LC–MS/MS method for the detection and quantification of ELA peptides in biological fluids and tissues. Using this method, we were able to identify the *in vitro* generated metabolites of ELA in human plasma and kidney. Interestingly, it was observed that like [Pyr^1^]apelin-13, ELA-32 was also cleaved proteolytically from both termini. In addition, attempts to use this assay to identify the most abundant endogenous isoform of ELA peptides present in human tissues, was mostly unsuccessful even using targeted MS/MS analysis.

The *in vitro* plasma half-life of ELA-32 observed in this study was approximately 47 min. This was consistent with pilot studies where peptide recovery from samples extracted in the absence of chaotropes was relatively low compared to when guanidine hydrochloride was present. Given the intensely charged nature of ELA-32 peptide, a plausible reason could be that the peptide was binding to plasma proteins to evade proteolytic degradation, but more studies are required to confirm this observation. It should be noted that the N-terminal Gln residue in this peptide was pyroglutaminated, which may have protected it from N-exoproteases [21]. However, a previous *in vitro* degradation study of [Pyr^1^]ELA-32 in rat plasma reported a half-life of ˜2 min [4], whereas the *in vivo* half-life of ELA-21 was suggested to be around 13 min [22] in mice. A similar discrepancy between *in vitro* plasma half-life in human and rodents were recently reported for [Pyr^1^]apelin-13 [23], thereby highlighting potential species differences in the repertoire of proteolytic enzymes. Additionally, the observed plasma half-life of ELA-32 in this study was significantly longer than that observed with apelin peptides, whose plasma half-life ranged from 2−8 min *in vitro* [4,24,25]. Therefore, of the two apelin receptor ligands, ELA may be the most resistant to proteolytic degradation.

Mature ELA-32 peptide has two potential furin cleavage sites characterised by di-arginine residues [26]. Based on this, the existence of two other isoforms of the ELA peptide, ELA-21 and ELA-11 were proposed [1,2]. Previous overexpression studies identified ELA-11 but not ELA-21 or ELA-32 [2], and more recently in a buffer-based ELA-32 digestion study, ELA-11 was identified as a furin metabolite [9]. ELA-21, which is one amino acid less than the expected furin cleavage site, has never been reported in degradation studies. Interestingly, apelin-55 also contain these di-arginine residues, and it was shown that proprotein convertase subtilisin/kexin 3 (PCSK3) cleaves immediately after these dibasic residues in the N-terminal to generate apelin-17 and apelin-13 [27,28]. Here, ELA-11 and ELA-22 were identified as fragments of ELA-32 generated *in vitro* in human plasma, but there was no evidence for the presence of ELA-21, either in the plasma or tissue homogenates. Consistent with our results, Murza et al., (2016) [4] observed the generation of ELA-22 but not ELA-21 in rat plasma *in vitro*. Taken together, these studies suggest a potential role of PCSK3 or other furin-like proprotein convertases in the cleavage of ELA-32 generating ELA-22 and ELA-11 but not ELA-21 in human plasma.

Other than furin, no other enzyme has been proposed to cleave ELA-32. ACE2 is well known for its ability to cleave apelin peptides, which have similar chemical properties and C-terminal amino acids to ELA peptides. ACE2 preferentially cleaves immediately after a proline residue in the C-terminal, and its activity has been demonstrated for many peptides, including angiotensin II, des-Arg^9^-bradykinin and apelin peptides [29,30]. Here, cleavage immediately after the terminal proline residue, resulting in the generation of ELA-16_1-14_ (CLQRRCMPLHSRVP|F) from des-Pro^16^-ELA-16 and ELA-19_1-17_ (KHNCLQRRCMPLHSRVP|F) from des-Pro^19^-ELA-19 were observed, which may suggest the activity of ACE2. These potential ACE2 substrates may have been generated from ELA-32_1-31_, which was the most abundant C-terminal metabolite identified. Importantly, these fragments are likely to be G protein-biased ligands of the apelin receptor, given that the loss of this phenylalanine residue impairs β-arrestin recruitment [4]. However, it is not clear whether, like bradykinin where an initial proteolytic removal of the C-terminal arginine (Arg^9^) residue is a prerequisite for ACE2 activity [29], the removal of C-terminal proline (Pro^32^) residue is a prerequisite for the observed ACE2 activity [8].

Several fragments of ELA-32, resulting from the loss of N-terminal amino acids were identified, including ELA-20 and ELA-16. Whether or not ELA-20 was generated from ELA-21, due possibly to the instability of the latter peptide in plasma, is unknown, but further interrogation of the data identified the corresponding 12 amino acid residues enzymatically removed from the N-terminus of ELA-20, suggesting that this was a primary cleavage site. ELA-16 and ELA-19 were generated much earlier, and ELA-16 was previously shown to have a similar affinity for the apelin receptor as ELA-32 [4]. Therefore, the ELA-16 fragment may be of potential importance as a tool compound for further studies as it contains a disulphide bridge in its N-terminal, which may stabilise it from proteolytic degradation. ELA-19 is novel and has not been reported to date.

It has previously been shown that ELA-32 fragments up to the last 11 amino acids at the C-terminus (ELA-11) were able to bind and activate the apelin receptor [7,31], suggesting that ELA-23, ELA-20, ELA-19, ELA-18, ELA-17, ELA-11 will have biological activity at the apelin receptor. Moreover, ELA-11 inhibited accumulation of cAMP with similar potency as ELA-32 and ELA-21 at the human apelin receptor but was less (˜10 fold) able to recruit β-arrestin when compared to the longer isoforms [4,7]. This may suggest that shorter fragments potently activate beneficial G protein signalling pathways with decreased ability to induce receptor desensitisation, thereby prolonging signalling activity. In support, ELA-14 also retained a subnanomolar affinity for the apelin receptor, reducing arterial pressure and exerting ionotropic effects on the heart both *ex vivo* and *in vivo* [4]. Similarly, recent studies suggest that ELA-11 was comparable to ELA-32 in its ability to prevent DNA damage-induced acute kidney injury [9] and suppression of tumour growth [32]. Collectively, these studies suggest that loss of N-terminal amino acids from mature ELA peptide does not adversely affect biological activity both *in vitro* and *in vivo*.

It remained unknown whether shorter ELA fragments like ELA-8 and ELA-9 identified in this study could bind and activate the apelin receptor, as with apelin, where shorter isoforms bind [30]. A previous structure-activity relationship (SAR) study of ELA peptides identified the four C-terminal amino acid residues His^26^, Arg^28^, Pro^30^ and Phe^31^ as critical for receptor binding [4]. Given that ELA-8 and ELA-9 retained these residues, it would be interesting to see whether they can bind the apelin receptor or act as receptor antagonists. Surprisingly, a recent SAR study suggested that ELA-9 had impaired binding affinity at the apelin receptor (K*_i_* = 438nM), whilst ELA-8 completely lost its ability to bind the apelin receptor and identified ELA-10 as the shortest active fragment of ELA. The authors also reported that the substitution of N-terminal methionine residue (Met^1^), from ELA-10 with the unnatural neutral amino acid, norleucine, resulted in ˜4 fold improvement in binding affinity [31]. Therefore, this may suggest that in addition to the amino acid residues previously reported to be important for binding, the three-dimensional structure of the peptide is critical to their ability to bind the apelin receptor. However, it remains to be determined whether these short fragments could be used as a backbone to develop novel ELA-base apelin receptor antagonists for experimental medicine since suitable antagonists at the apelin receptor are lacking.

The identification of ELA-32_(1-21)_, ELA-32_(1-20)_ and ELA-32_(1-19)_ may suggest a sequential proteolytic cleavage of ELA-32 from the C-terminal. Interestingly, except ELA-32_(1-20)_ and ELA-32_(1-19)_ whose corresponding N-terminal metabolite was not found, the corresponding N-terminal metabolites of these fragments were identified. Additionally, ELA-32_(1-12)_, ELA-32_(1-13)_, ELA-32_(1-14)_ and ELA-32_(1-16)_ resulting from the loss of C-terminal amino acids could be detected. Notably, these fragments are unlikely to have biological activity at the apelin receptor since the critical pharmacophore required for binding are present in the C-terminal, which has been removed [4]. Conversely, for [Pyr^1^]apelin-13, the RPRL motif critical for apelin receptor binding is present on the N-terminal [6,33,34]. Therefore, it appears that these peptides (apelin and ELA) evolved in such a way that any enzymatic degradation preserves their pharmacophore.

The most predominant endogenously expressed isoform of ELA in mammals remains unknown. Following its discovery, Chng et al., (2013) [1] reported the identification of mature ELA-32 in zebrafish using a specific N-terminal antibody. Similarly, after overexpression of ELA mRNA expression in zebrafish, Pauli et al., 2014 [2] reported the identification of ELA-11 by mass spectrometry. Conventional immunological methods are unable to distinguish between the various isoforms; therefore, using mass spectrometry, we sought to identify, for the first time *in vivo,* the most abundant ELA isoform. Initial studies on human kidney and coronary artery failed to detect any ELA isoform in these tissues. In order to enrich for ELA peptides following tissue extraction, ELA peptides were immunoprecipitated before mass spectrometry, but the peptides were still undetectable. A targeted MS/MS approach was also investigated, but ELA peptides where undetectable down to 100 pg/mL (ELA-11) and 500 pg/mL (ELA-21 and ELA-32), suggesting that if present in the tissues examined, their endogenous concentration was below our limit of detection.Levels of endogenous ELA peptides could be quantified in human kidney, coronary artery and brain samples using the ELISA. However, the presence of multiple arginine residues in the ELA molecule resulted in column retention and poor chromatographic separation by mass spectrometry. The addition of TFA to the mobile phase improved the peak profile but resulted in decreased sensitivity [35,36] and precluded peptide detection. Therefore, currently, the only robust method for measurement of endogenous ELA is the ELISA, although this method does not distinguish between the different ELA isoforms.

In conclusion, we have shown that ELA-32 may be the most stable of the apelin receptor endogenous ligands, apelin and ELA, with an *in vitro* half-life of approximately 47 min in plasma. In addition, several biologically active metabolites of ELA-32 were identified, notably ELA-16 and ELA-19 or ELA-22. Considering that ELA-16 has a di-sulphide bridge in its N-terminal, it may be the most stable isoform of the peptide that retains activity at the apelin receptor. Attempts to identify the endogenous levels of these peptides proved difficult owing to their physicochemical properties, which predispose ELA peptides to oxidation and poor chromatographic peaks. Given the beneficial effects of ELA signalling in various disease states including renal [9,37,38,8,32,33] and cardiovascular diseases [6,39], the long half-life observed here may suggest that ELA peptides could be a potential therapeutic option for managing these conditions.

## Grant information

We thank the following for full or partial support: Wellcome Trust (United Kingdom), (WT107715/Z/15/Z, APD, JJM); Wellcome Trust (United Kingdom), Programme in Metabolic and Cardiovascular Disease (203814/Z/16/A, DN), British Heart Foundation (United Kingdom), TG/18/4/33770, Cambridge Biomedical Research Centre Biomedical Resources (United Kingdom) Grant (University of Cambridge, Cardiovascular Theme, RG64226). We thank the Royal Papworth Hospital Research Tissue Bank, Human Research Tissue Bank and Cambridge Brain Bank who are supported by the NIHR Cambridge Biomedical Research Centre, (United Kingdom). The views expressed are those of the author(s) are not necessarily those of the NIHR or the Department of Health and Social Care.

## CRediT authorship contribution statement

**Duuamene Nyimanu:** Methodology, Investigation, Formal analysis, Writing - original draft, Writing - review & editing. **Richard G. Kay:** Methodology, Investigation, Formal analysis, Writing - original draft, Writing - review & editing. **Rhoda E. Kuc:** Methodology, Investigation, Formal analysis, Writing - original draft, Writing - review & editing. **Alastair J.H. Brown:** Conceptualization, Methodology. **Fiona M. Gribble:** Resources, Writing - review & editing. **Janet J. Maguire:** Conceptualization, Supervision, Methodology, Investigation, Formal analysis, Writing - original draft, Writing - review & editing, Funding acquisition. **Anthony P. Davenport:** Conceptualization, Supervision, Methodology, Investigation, Formal analysis, Writing - original draft, Writing - review & editing, Funding acquisition.
