# Supplementary material for: In vitro metabolism of synthetic Elabela/Toddler (ELA-32) peptide in human plasma and kidney homogenates analyzed with mass spectrometry and validation of endogenous peptide quantification in tissues by ELISA
Source: Peptides. 2021 Nov;145:170642. doi: 10.1016/j.peptides.2021.170642 (PMC8484864; doi:10.1016/j.peptides.2021.170642)
Supplement: Supplementary file 1 [file mmc1.docx]

**Supplementary Information**

Results


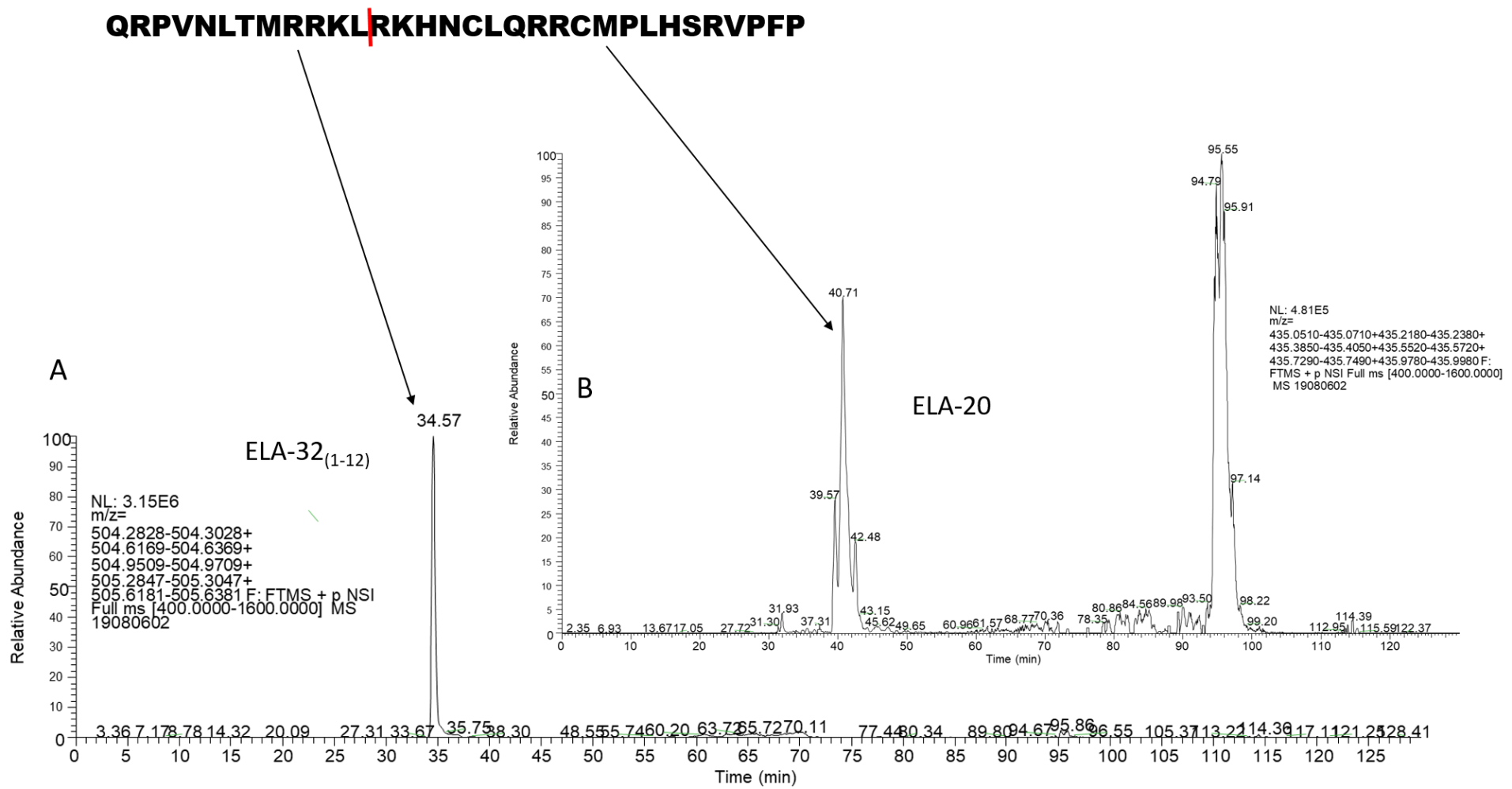


Supplementary Figure 1A. Chromatogram showing ELA-32_(1-12)_ (A) and its C-terminal fragment ELA-20 (B). ELA-32_(1-12)_ is unlikely to be able to bind the apelin receptor because the critical pharmacophores required for binding are in the C-terminus (ELA-20).


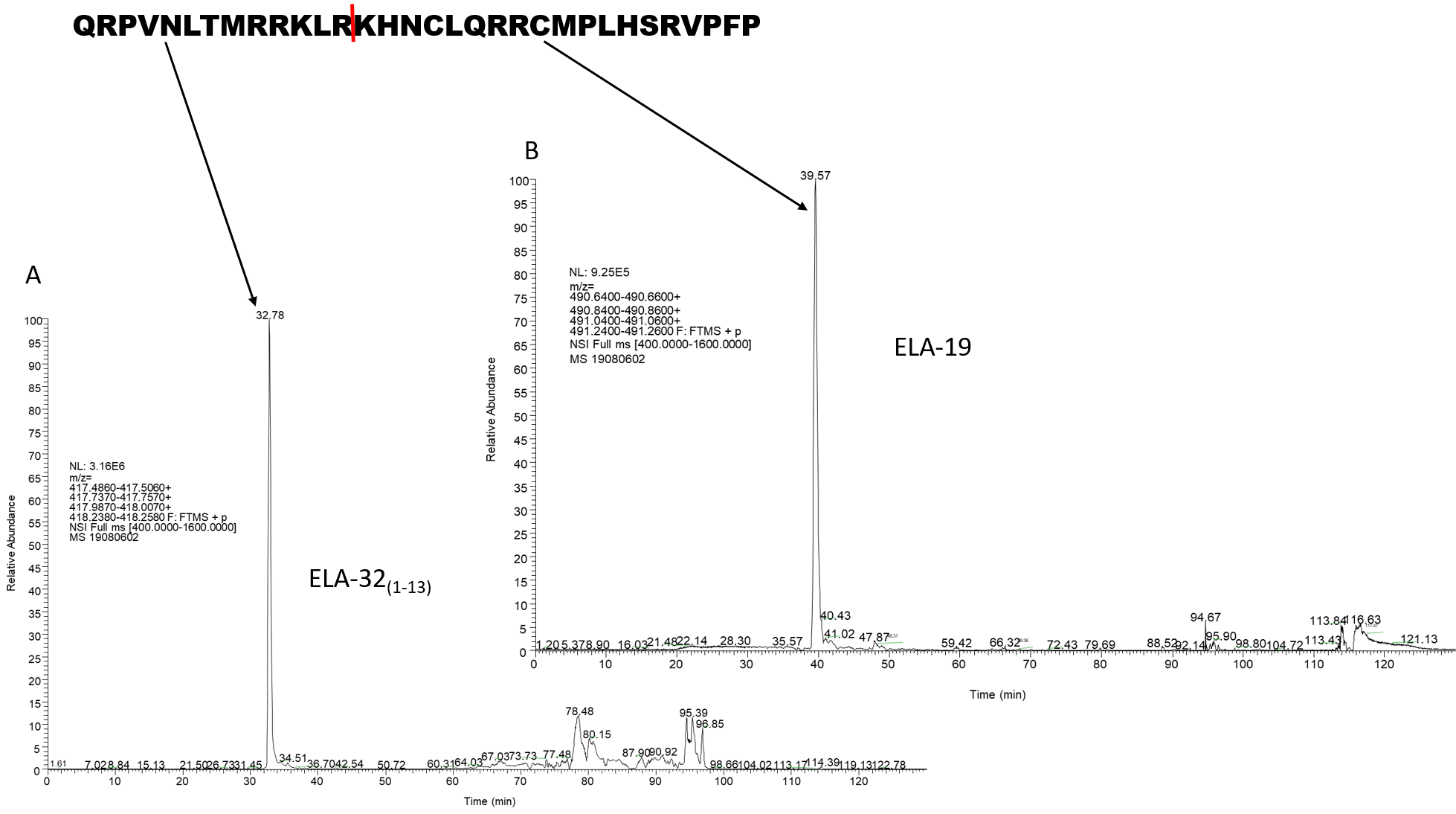
Supplementary Figure 1B. Chromatogram showing ELA-32_(1-13)_ (A) and its C-terminal fragment ELA-19 (B). ELA-32_(1-13)_ is unlikely to be able to bind the apelin receptor because the critical pharmacophores required for binding are in the C-terminus (ELA-19).


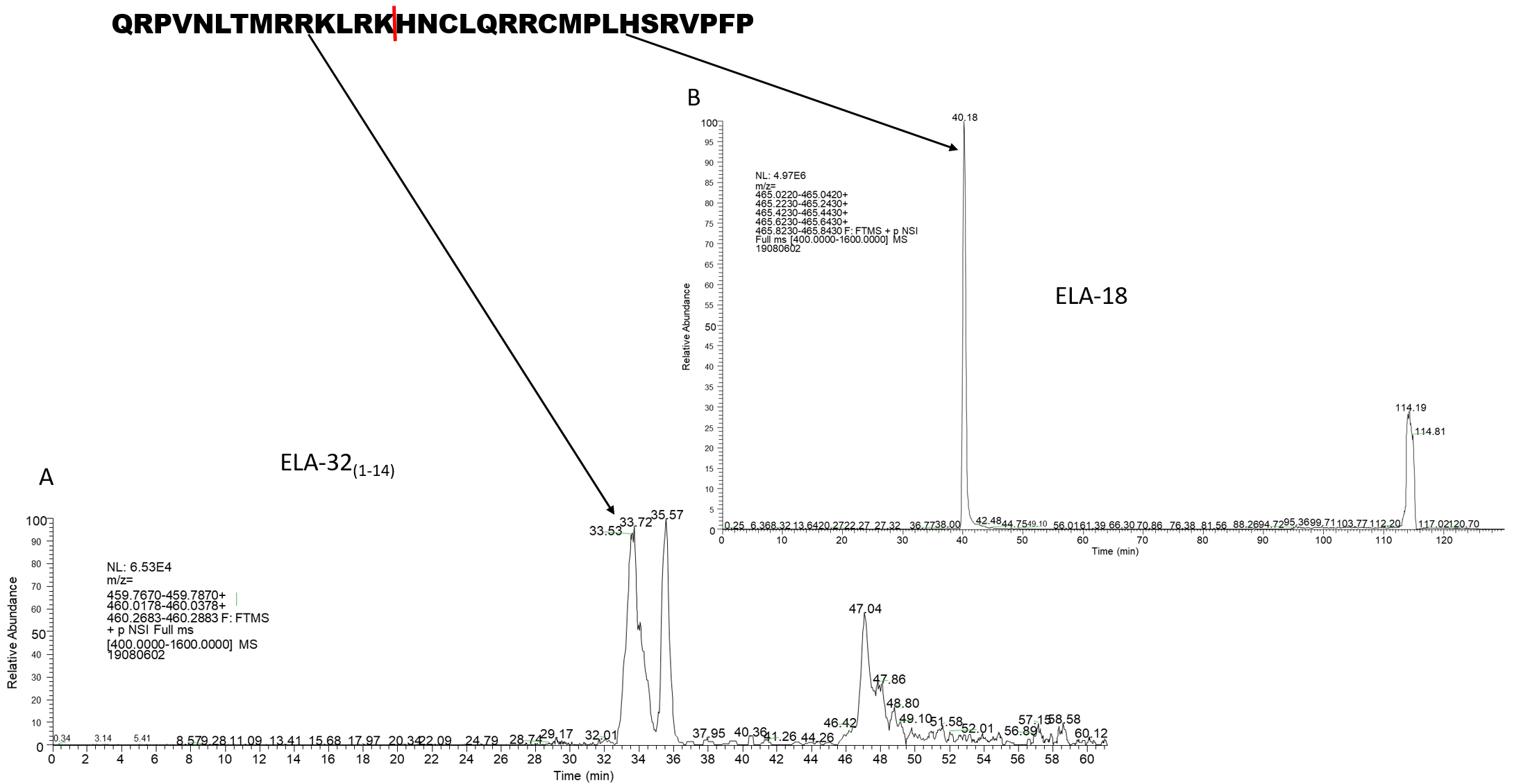
Supplementary Figure 1C. Chromatogram showing ELA-32_(1-14)_ (A) and its C-terminal fragment ELA-18 (B). ELA-32_(1-14)_ is unlikely to be able tobind the apelin receptor because the critical pharmacophores required for binding are in the C-terminus (ELA-18). This ELA-18 was
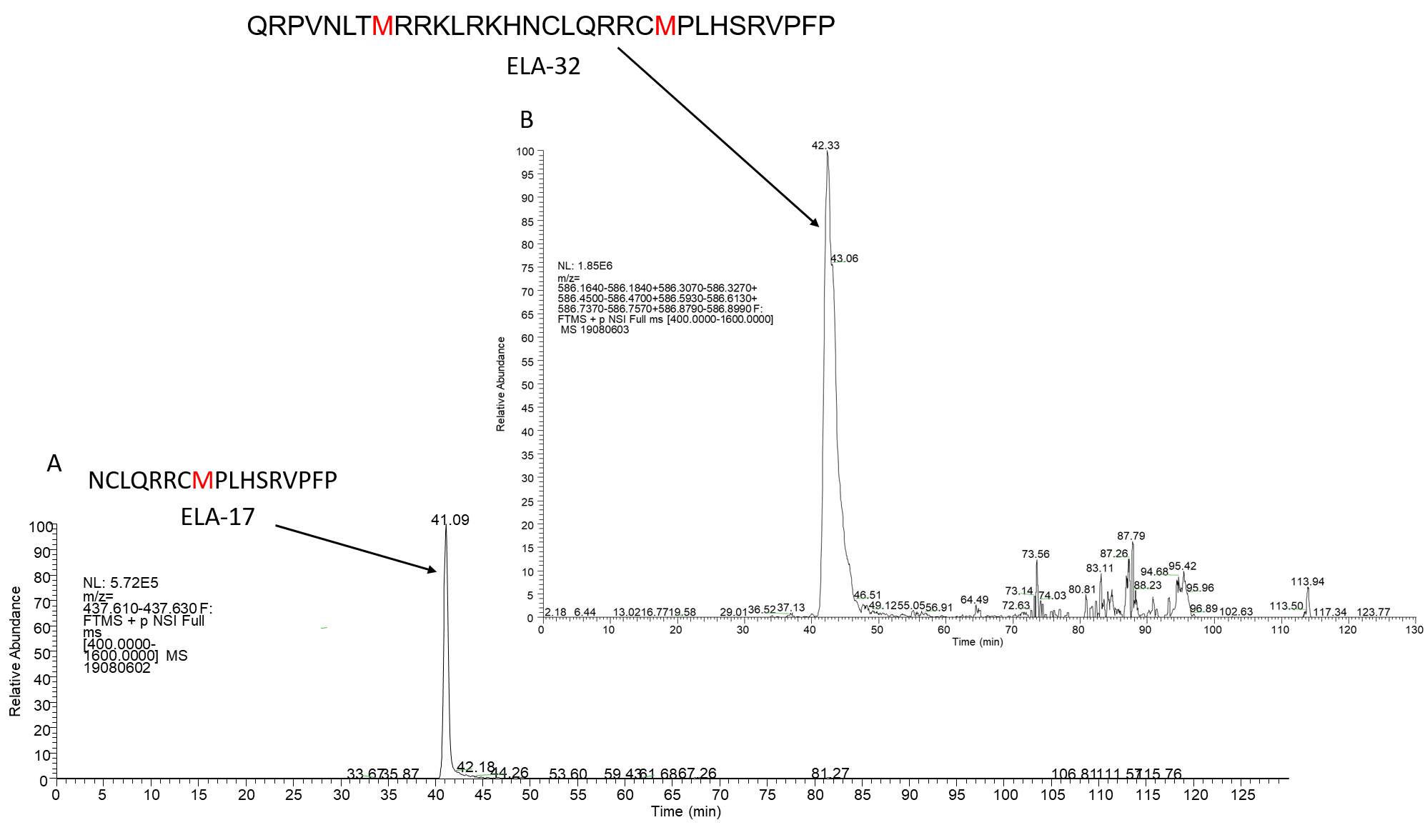
oxidised on the methionine residue.

Supplementary Figure 1D. Chromatogram showing ELA-32 (B) and its C-terminal fragment ELA-17 (B). ELA-32 shown is the dioxidised form
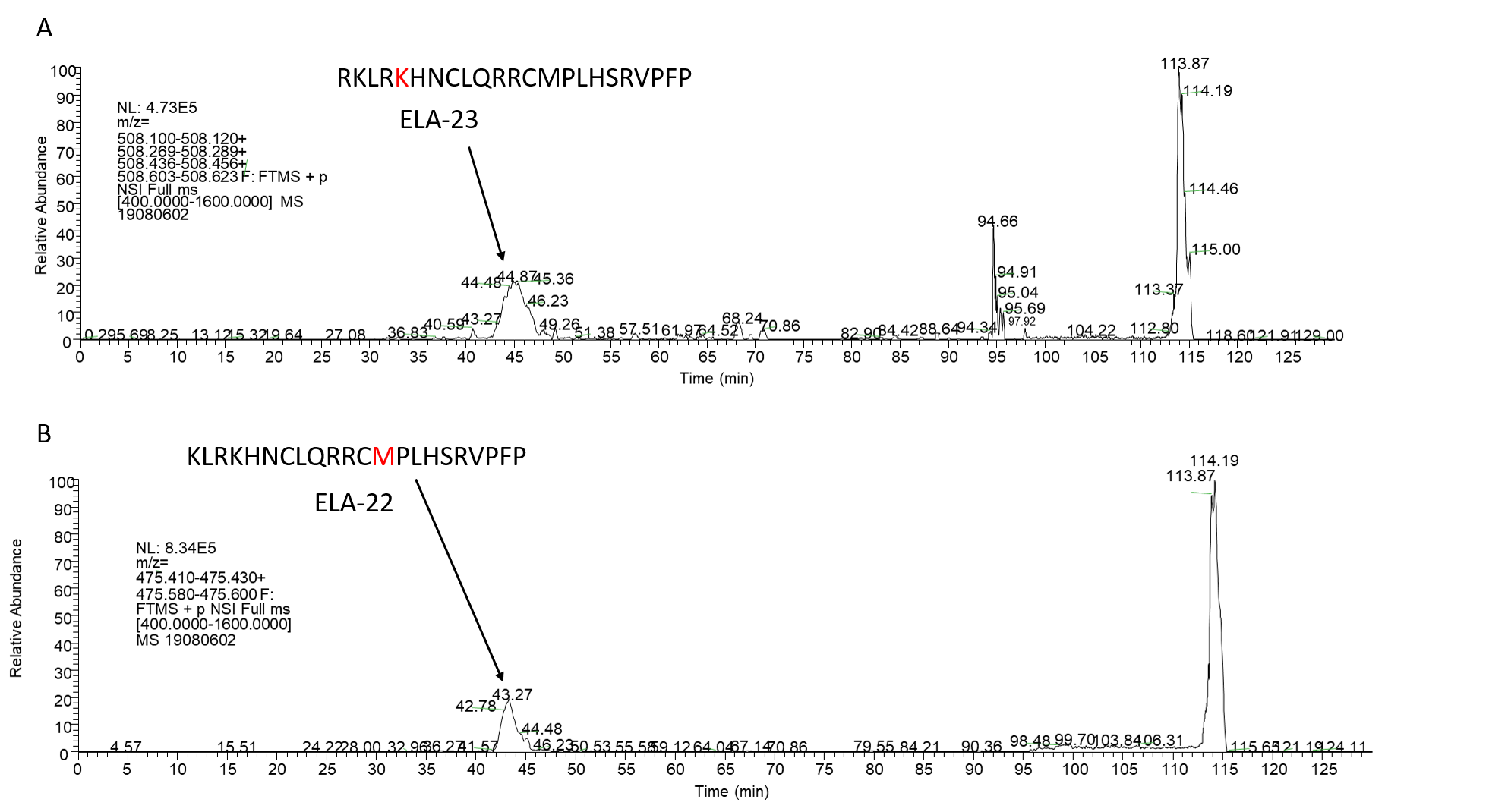
where both methionine residues are oxidised. ELA-17 is also oxidised on its methionine residue.

Supplementary Figure 1E. Chromatogram showing ELA-23 (A) and ELA-22 (B). ELA-23 was alkylated on its lysine residue shown in red while ELA-22 was oxidised on the methionine residue. Both of these isoforms are likely to be functional.


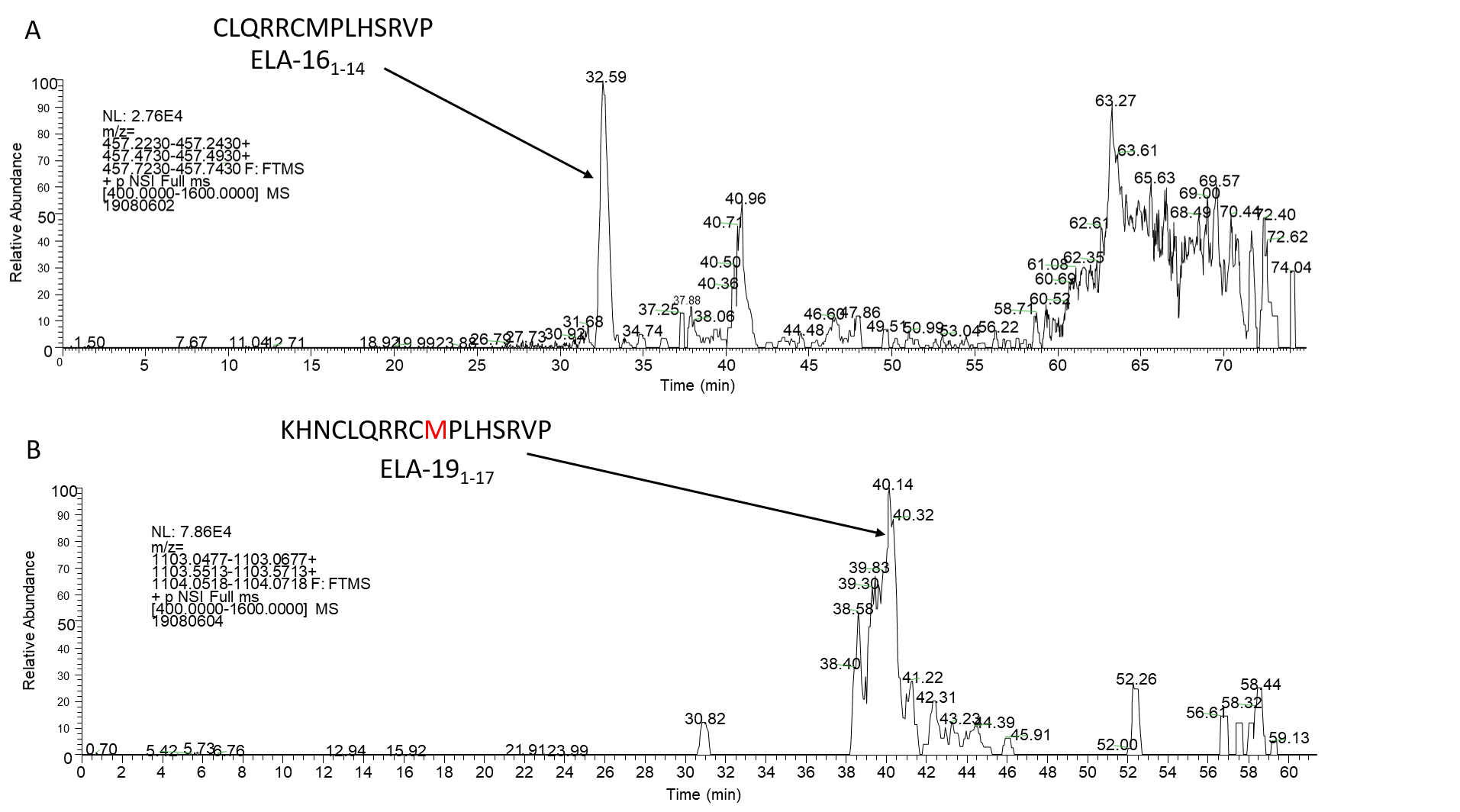
Supplementary Figure 1F. Chromatogram showing ELA-16_(1-14)_ (A) and ELA-19_(1-19)_ (B) derived from des-Pro^17^-ELA-19 and des-Pro^16^-ELA-16 respectively. These fragments may have been generated by ACE2 activity.


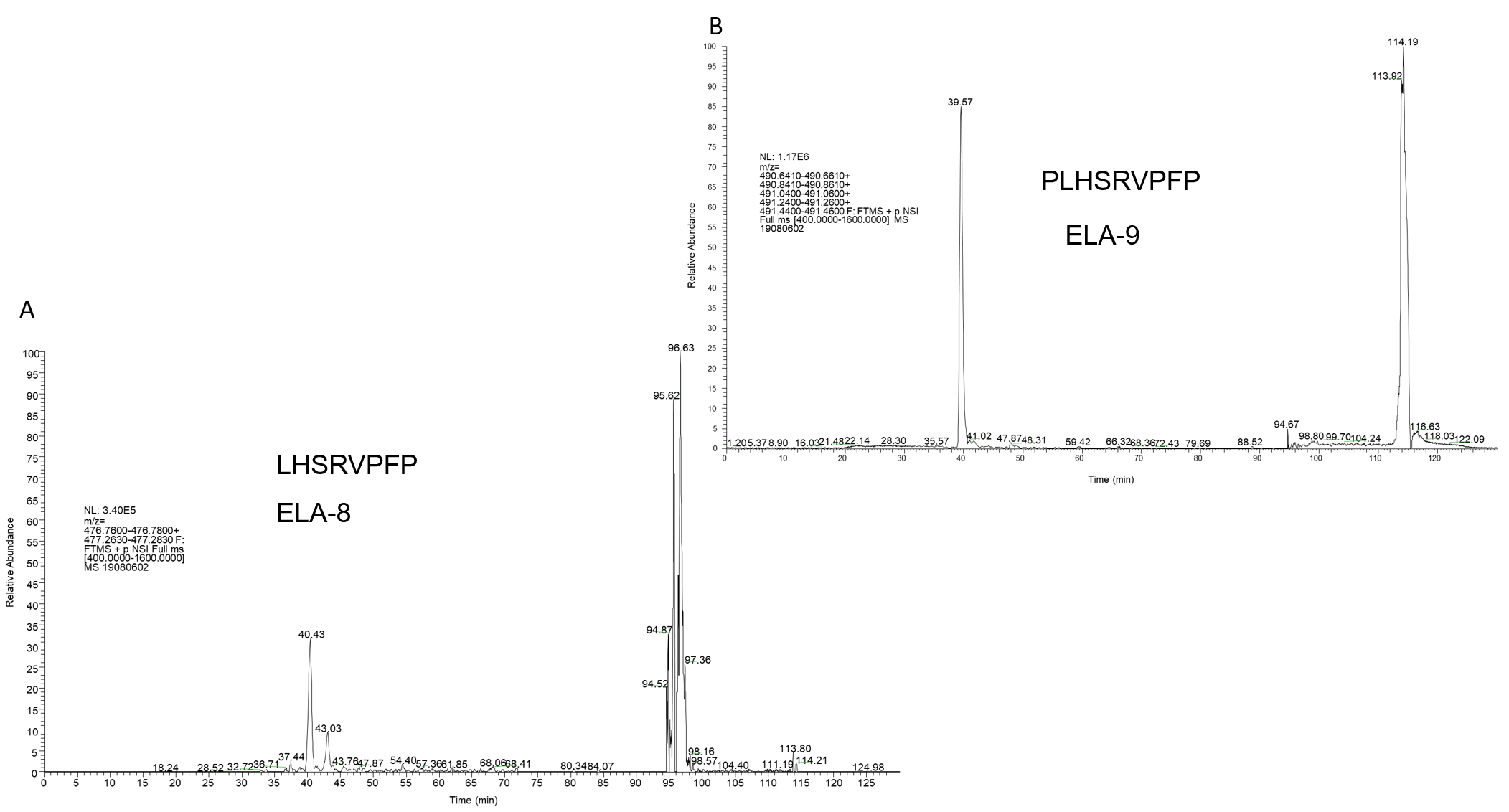


Supplementary Figure 1G. Chromatogram showing ELA-8 (A) and ELA-9 (B). It is not clear whether these fragments would bind the apelin receptor although they contain the critical pharmacophores required for binding.

***Enrichment of ELA peptides in extracted samples by Immunoprecipitation***

Antibody based enrichment of peptide of interest following extraction is a well characterised method for concentration of analytes in solution [20]. Therefore, in order to concentrate ELA peptides following extraction from human tissues, polyclonal primary antibody raised against ELA-32 peptide, but which cross-reacts with ELA-21 and ELA-11 was used. The affinity of the antibody for all three isoforms were assessed using two types of beads, Dynabeads M-280 tosylactivated and Protein A/G magnetic beads coupled to either purified or unpurified antibodies at two different concentrations, 5ng/mL and 250ng/mL representing lower and upper limits. It was observed that Protein A/G coupled to unpurified anti-ELA antibody appeared to perform better at capturing ELA peptides from solution (Sup. Fig. 1). Hence, further studies were continued using Protein A/G magnetic beads and unpurified antibody.

*
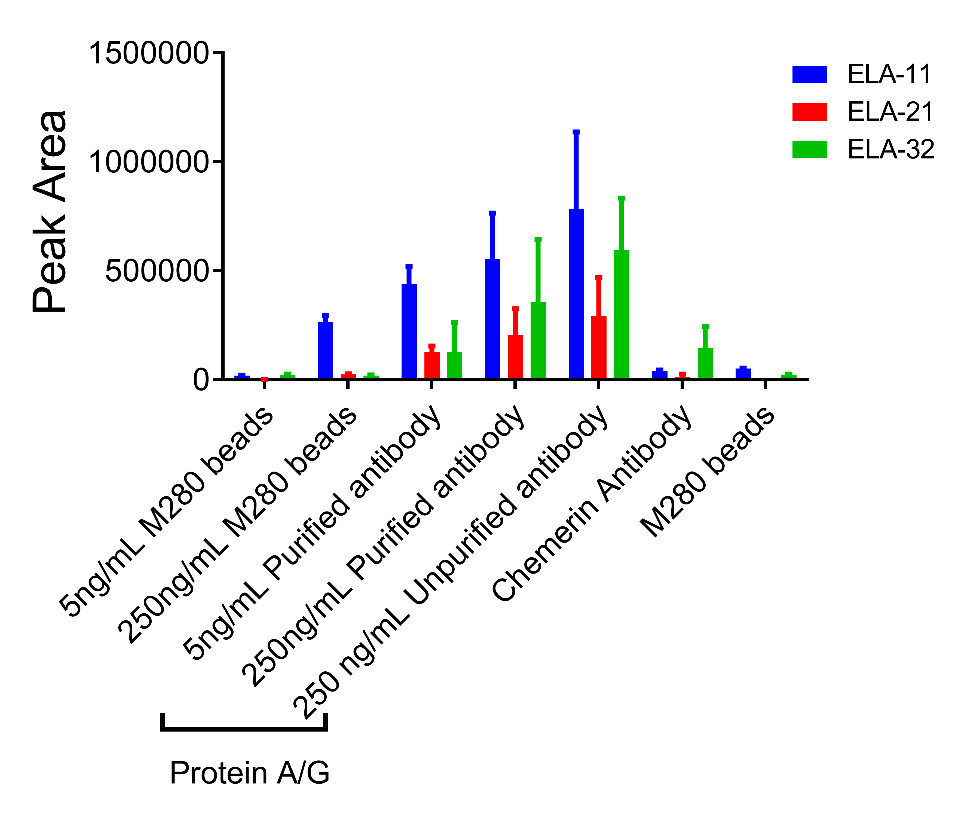
*

*Supplementary figure 2. Validation of antibody affinity for the ELA isoforms. Antibody coated protein A/G or Dynabeads M-280 tosylactivated were added to solution containing ELA-11, ELA-21 and ELA-32 at the indicated concentrations (5ng/mL, or 250ng/mL). Immunoprecipitated peptides were analysed on mass spectrometry. Data represent Mean±SEM.*
